# Carbon emissions of power transmission and transformation projects in the whole life cycle for smart sustainable energy systems

**DOI:** 10.1038/s41598-024-54317-0

**Published:** 2024-02-15

**Authors:** Zhihui Wang, Long Hu, Xiaojia Huang, Jieren Tan, Kaihui Ye

**Affiliations:** 1 Guangzhou Power Supply Bureau, Guangdong Power Grid, Co., Ltd., CSG, Guangzhou, 510000 People’s Republic of China; 2 Guangdong Electric Power Design Institute Co., Ltd., Guangzhou, 510000 People’s Republic of China

**Keywords:** Energy science and technology, Energy infrastructure, Energy grids and networks

## Abstract

The study investigates the optimization of life cycle carbon emissions in smart sustainable energy systems through power transformation and transmission project power load predictions. Firstly, a multi-task learning-based short-term user load forecasting technique is developed, where the power load curves of multiple residential customers are grouped and classified using the K-means clustering method. Additionally, the Bidirectional Long Short-Term Memory (BiLSTM) technique is introduced to anticipate the power load intelligently. Secondly, a life cycle carbon emission assessment model for the power transmission and transformation project (PTTP) is constructed based on the life cycle assessment (LCA) method, which divides the project's life cycle into four stages: production, installation and construction, operation and maintenance, and demolition. Finally, an experimental evaluation of this model is conducted. The results demonstrate that compared with the baseline model Long Short-Term Memory (LSTM), this model achieves a significantly lower average Mean Absolute Error (MAE) at 3.62% while achieving significantly higher accuracy in power load forecasting at 94.34%. A comprehensive examination of carbon emissions across all four phases reveals that overall carbon emissions are highest during the operation and maintenance stage followed by the equipment production stage and installation/construction stage, with the lowest overall carbon emissions observed. Hence, this study endeavors to forecast power load demand with precision and identify the principal determinants of carbon emissions in power engineering. By discerning and managing these key factors, an optimal, energy-efficient intelligent power load scheme can be derived.

## Introduction

In the contemporary global context, anthropogenic actions, including the combustion of fossil energy, deforestation, and extensive agricultural practices, have given rise to substantial emissions of greenhouse gases. Consequently, there has been a persistent escalation in the concentration of greenhouse gases within the atmosphere. Climate change has exacerbated the frequency and intensity of natural disasters such as floods, droughts, and hurricanes, which have resulted in crop failures and harmed the livelihoods of farmers. Impoverished regions often lack sufficient resources and infrastructure to cope with these disasters, rendering their resilience more fragile and intensifying issues of poverty. Simultaneously, climate change has precipitated environmental changes, including heightened air pollution and increased occurrences of heatwaves, posing a direct threat to human health. According to the World Health Organization, millions of people worldwide suffer from heat-related illnesses annually, with approximately 20% of cases resulting in fatalities^1^. Owing to the exigencies of climate change and the imperative for sustainable energy, the energy industry is expediting the shift towards renewable and clean energy sources, encompassing solar and wind energy, as well as other forms of zero-emission energy^2,3^. Within this transformative paradigm, Power Transmission and Transformation Projects (PTTP) assume a pivotal role in ensuring the dependable distribution and integration of renewable energy throughout the entire energy system^4^. However, the processes of constructing, operating, and maintaining PTTP inherently give rise to carbon emissions, prompting significant concern against the backdrop of contemporary imperatives for sustainable development and the imperative to reduce carbon emissions (referred to as “double carbon”)^5^.

Therefore, as a crucial component of the energy system, what specific magnitude does the carbon emissions of the entire life cycle of the power transmission and transformation (PTT) engineering occupy? Addressing this question involves positing hypotheses about the potential adverse environmental impacts of carbon emissions during the construction and operation phases of PTT projects^6,7^. Subsequent derived hypotheses suggest that within the hierarchical arrangement of carbon emissions throughout the life cycle of PTT engineering, the total carbon emissions during the operation and maintenance phase are the highest, followed by the equipment production phase, with the installation and construction phase exhibiting the lowest total carbon emissions. Addressing these inquiries and hypotheses becomes imperative for investigating the carbon emissions of PTT engineering over its entire life cycle under the context of low-carbon environmental benefits. Such an exploration holds significant practical significance for the development and construction of intelligent and sustainable energy systems.

Mitigating carbon emissions stands as a collective challenge confronting global society. Within the realm of carbon-emitting sources, the power system holds the potential to contribute to the development of sustainable energy systems and alleviate its deleterious impact on the climate through a comprehensive understanding of the carbon emissions emanating from PTTP throughout their entire life cycle. Positioned as a highly integrated and intelligent energy infrastructure, the intelligent sustainable energy system aspires to furnish a clean, sustainable, efficient, and reliable energy supply^8,9^. This system leverages advanced technology and intelligent control methodologies to diminish reliance on traditional fossil fuels; nonetheless, the smart grid itself generates greenhouse gases, underscoring the importance of carbon emission assessment in elucidating the greenhouse gas content within the environment. Life Cycle Assessment (LCA) emerges as a systematic approach for evaluating the environmental impact of products, processes, or systems, with one of its central objectives being the assessment of carbon emissions. Broadly categorized, LCA encompasses process-based LCA, economic input–output LCA, and hybrid LCA^10^. Typically, unfolding across four principal stages—inventory, assessment, interpretation, and improvement. LCA involves data collection and modeling during the inventory phase, environmental impact analysis during the assessment phase, result elucidation during the interpretation phase, and formulation of improvement recommendations based on the evaluation outcomes during the improvement phase^11,12^. Numerous scholars have conducted pertinent research on the current status of energy transition and carbon emissions, as outlined in Table 1.

**Table 1:** Compilation of literature on intelligent energy transition and carbon emissions.

Scholar	Research methodology	Role or effect
Murshed et al.^13^	They investigated the role of renewable energy transition and global trade	This approach underscored the critical role of renewable energy, emphasizing the importance of international trade cooperation and providing crucial insights for achieving carbon neutrality goals
Bouyghrissi et al.^14^	They focused on Morocco's emission reduction goals, particularly emphasizing the significance of renewable energy transformation	Results demonstrated that the shift toward renewable energy was a primary factor in reducing carbon dioxide emissions
Bacanin et al.^15^	They employed a metaheuristic algorithm to optimize the energy load prediction of deep learning models	The method pointed out the potential for enhancing prediction accuracy, offering a more effective tool for energy management
Vakulchuk et al.^16^	They examined strategies to attract more investments in renewable energy	The approach highlighted the pivotal role of renewable energy in regional sustainable development
Rinne et al.^17^	They compared the LCA and carbon footprints of concrete and wooden residential buildings in Finland	The method provided important insights into the environmental impact of different building types, aiding decision-makers in better understanding the significance of sustainable building choices
Joensuu et al.^18^	They discussed LCA methods for buildings in the context of a circular economy	The approach held significant implications for promoting sustainability and resource utilization efficiency in the field of architecture
Lu et al.^19^	They utilized dynamic LCA methods to analyze the carbon footprint of future solar energy in the United States	The method offered key information for the sustainability and environmental impact of solar energy
Zhang et al.^20^	They conducted an economic analysis of hydrogen production in provincial-level power grids in China	The approach facilitated the transformation of power systems, considering the impact of carbon emissions
Yang et al.^21^	They proposed carbon emission improvement methods for independent energy production within the power grid context	This method provided a foundation for accurately assessing and managing the carbon footprint of power systems

In summary, through an analysis of the studies by the aforementioned scholars, it is evident that while there have been significant advancements in research on energy transition, challenges still persist. These challenges include issues related to energy storage, the reliability of energy supply, and the practical implementation of carbon emission reduction. Moreover, in carbon emission research, more scholars have focused on the entire lifecycle of carbon emissions of buildings, with very few extending these studies to the field of electrical engineering. Therefore, the innovation of this study lies primarily in its comprehensive and in-depth exploration of carbon emissions in power transmission projects within smart sustainable energy systems. Firstly, the study introduces the K-means clustering and Bidirectional Long Short-Term Memory (BiLSTM) algorithm, designing a short-term user load forecasting scheme based on multi-task learning. Secondly, a carbon emission assessment of each phase of power transmission projects is conducted using the LCA method, revealing the highest carbon emissions during the operational maintenance phase, followed by the equipment production phase, and the lowest carbon emissions during the installation and construction phases. Finally, the model's performance is experimentally evaluated to validate its effectiveness. This study provides robust methods and strategies for achieving intelligent and energy-efficient electricity load schemes. Additionally, it offers crucial guidance and references for understanding and controlling the key factors influencing carbon emissions in power transmission projects.

The following is how the remaining portions of this study are organized: “Method” focuses on the research methodology, encompassing the construction of a power demand forecasting scheme for power transmission projects within smart sustainable energy systems, as well as the development of a lifecycle-based carbon emission calculation model for power transmission projects. The section also includes an evaluation and analysis of the performance of the constructed models. “Results and discussion” presents the experimental results and discussions, offering a detailed analysis of both the model's performance and the outcomes of the experiments. “Conclusions and prospects” serves as the conclusion, summarizing the key findings of this study and outlining potential directions for future studies.

## Method

In this section, for the transmission and transformation projects in the intelligent sustainable energy system, the intelligent algorithm is first introduced to build the power demand forecasting scheme, and then the lifecycle-based carbon emission calculation model is established. These methods are designed to understand the role of transmission and transformation projects in sustainable energy systems, as well as the carbon emissions of their whole life cycle. The performance evaluation analysis of the model will help evaluate its reliability and applicability.

### Power transformer analysis in intelligent sustainable energy system

Sustainable energy pertains to energy sources capable of satisfying present demands without compromising the ability of future generations to meet their own needs. Such sources are characterized by their capacity to curtail carbon emissions throughout energy production and consumption processes, exhibiting minimal environmental impact and the potential for continuous provision^22,23^. The categorization of these sources is presented in Table 2.

**Table 2:** Classification table of sustainable energy.

Type	Power generation mode	Advantage	Common production capacity range
Solar energy	Solar radiation is converted into electricity or heat energy by solar panels or solar thermal energy systems	The unlimited solar energy supply and low carbon emission are suitable for various applications	1–10 kW/m^2^
Wind energy	Through a wind turbine, the blades of the turbine are rotated by wind to generate electricity	The unlimited wind energy supply and low carbon emission are suitable for large-scale and distributed systems	1–5 MW/turbine
Waterpower	Power is generated by driving a hydraulic turbine by water flow	It is efficient, renewable, reliable, and suitable for various terrains and scales	1–100 MW/dam
Geothermal energy resources	It rises from the underground hot rock layer by hot water or steam and then generates electricity by steam turbine	It is sustainable and low-carbon emission, suitable for tropical and volcanic areas in specific areas	1–10 MW/well
Biomass energy	Electricity is generated by burning, fermenting, or gasifying organic materials	Renewable and widely available raw materials are helpful for waste management	1–20 MW/biomass plant

**Figure 1:**
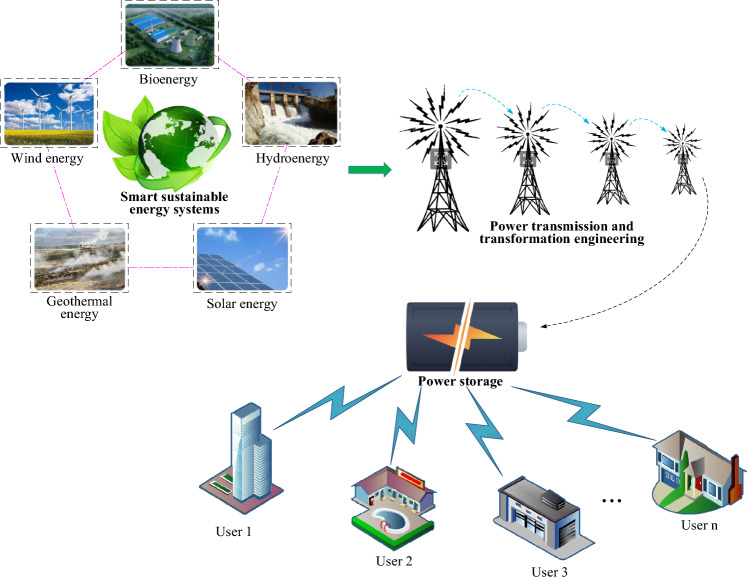
Schematic diagram of PTTP of the intelligent sustainable energy system.

The electricity produced through sustainable energy sources is subsequently conveyed to the intelligent, sustainable energy system. The PTTP assumes the responsibility for transmitting renewable energy-generated power to the consumption destination, ensuring the dependable distribution and integration of energy, as illustrated in Fig. 1.

In Fig. 1, within the intelligent sustainable energy system, the PTTP achieves real-time monitoring and management of the power system through intelligent sensors, communication systems, and data analysis. All components within the PTTP must collaborate synergistically to facilitate the seamless distribution of renewable energy^24^. Notably, artificial intelligence algorithms are instrumental in predicting power demand, coordinating renewable energy supply, managing energy storage systems, and harmonizing the operation of diverse power grid segments to ensure overall balance and security.

### Power demand forecast and analysis of PTTP

**Figure 2:**
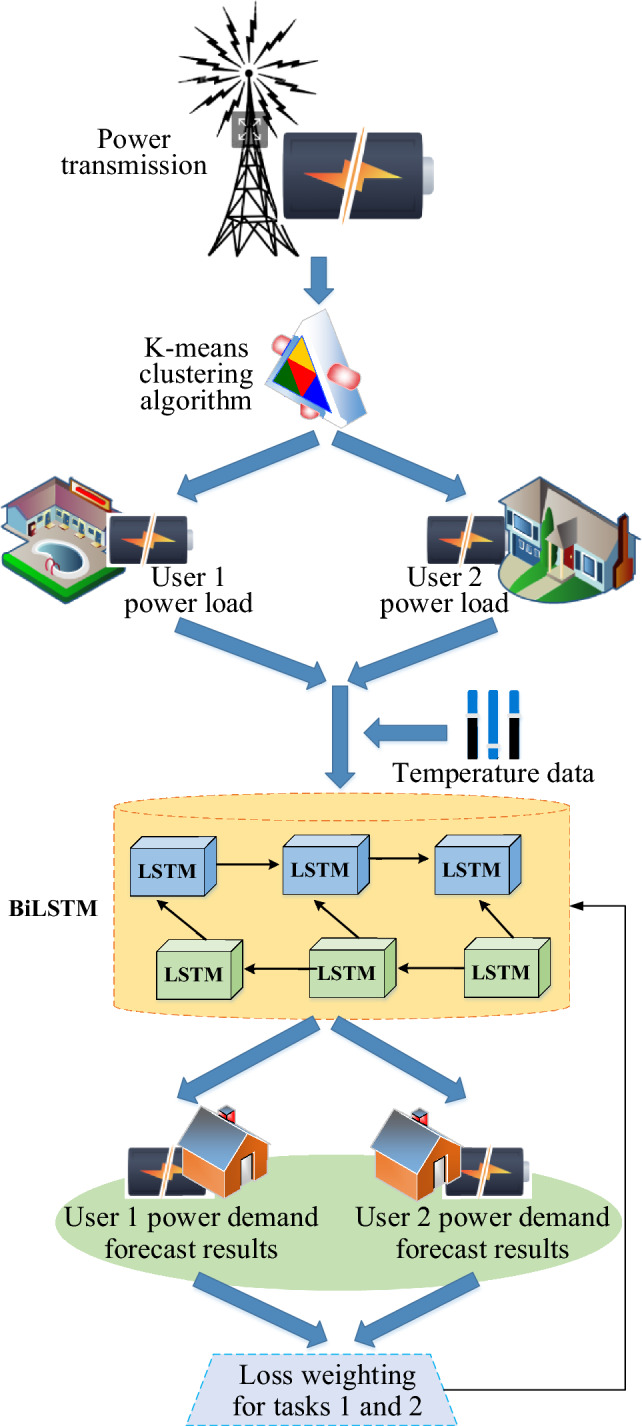
Flow chart of short-term user load forecasting scheme based on multi-task learning.

The PTTP incorporates a deep learning algorithm to forecast power demand, optimize the operation of various components, maintain power system equilibrium and safety, and enhance the efficiency, reliability, and sustainability of the PTTP. This initiative aims to mitigate the pronounced fluctuations and uncertainties arising from residents' behaviors impacting total load^25^. Consequently, the study advocates for the implementation of a multi-task learning-based short-term residential load forecasting system, as illustrated in Fig. 2.

In Fig. 2, the depicted process comprises two primary stages: the allocation stage and the prediction stage. In the allocation stage, the initial step involves the utilization of the K-means clustering algorithm^26,27^ to cluster power load curves from a substantial number of residential users. Subsequently, within each cluster, two residential users exhibiting comparable power load curves are selected, facilitating the grouping of users for subsequent power load forecasting.

During the forecasting stage, a time series is formulated by integrating forthcoming temperature data with the historical power load data of the chosen pair of customers. Following this, a Bidirectional Long Short-Term Memory (BiLSTM) model processes the amalgamated sequence^28^. Ultimately, the outputs of the forward and reverse LSTM networks are consolidated within the BiLSTM network and directed to the designated output layers for each of the two tasks, typically involving temperature and power load anticipation. In order to ensure the network optimally addresses both tasks concurrently, the network's loss function is a weighted sum of the loss functions associated with the two tasks.

In clustering consumers' electrical usage, the Pearson correlation coefficient serves as a commonly employed metric to ascertain the level of correlation between two variables. Equation (1) illustrates the application of this method in calculating the correlation degree between the load curves of two users.

1 $$ r_{xy} = \frac{{\sum\nolimits_{i = 1}^{n} {x_{i} y_{i} } - n\overline{x}{\kern 1pt} \overline{y}}}{{\left( {n - 1} \right)S_{x} S_{y} }} $$

$x_{i}$ and $y_{i}$ refer to the load values of the load curves of two users at the ith hour. $\overline{x}$ and $\overline{y}$ refer to the average values of the load curves of two users, *n* refers to the total hours of the load curves of two users. $S_{x}$ and $S_{y}$ refer to the standard deviations of the load curves of two users. In this study, according to the numerical range grade of Pearson coefficient used by Beerwinkle et al. (2021)^29^, the range grade of Pearson coefficient is specified as shown in Table 3:

**Table 3:** Pearson coefficient numerical domain hierarchy table.

Numerical value	r_xy_ > 0.8	0.6 < r_xy_ ≤ 0.8	0.4 < r_xy_ ≤ 0.6	0.2 < r_xy_ ≤ 0.4	0 < r_xy_ ≤ 0.2
Correlation between two curves	Extremely strong correlation	Strong correlation	Moderate correlation	Weak correlation	Very weak correlation or irrelevance

### Carbon emission analysis of lifecycle assessment applied to PTTP

**Figure 3:**
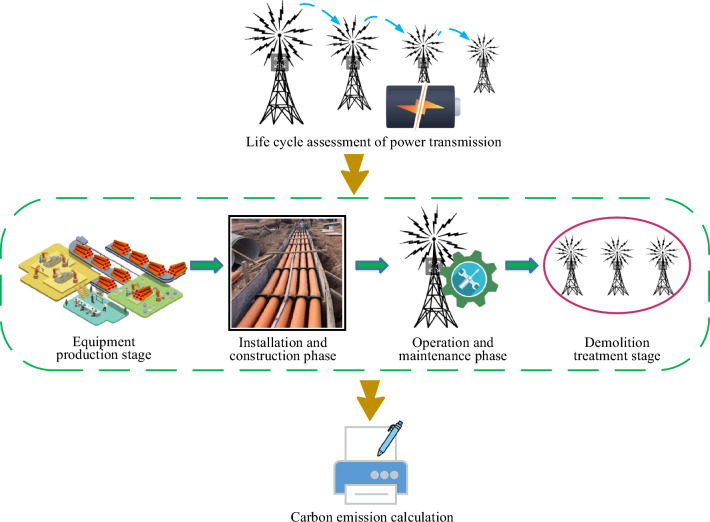
Life cycle carbon emission flow chart of PTTP based on LCA.

Following the allocation of power load based on demand within the PTTP, a subsequent analysis of carbon emissions in the PTTP is conducted through the employment of the LCA method. This study delineates the life cycle of the PTTP within an intelligent sustainable energy system into four distinct phases: the production stage, installation stage, operation and maintenance stage, and demolition stage. The carbon emissions calculation framework throughout its life cycle is illustrated in Fig. 3.

In the computation of the carbon emissions of the PTTP employing the LCA method, the production stage of power pipeline equipment involves the calculation of CO_2_ emissions arising from raw material extraction, transportation, and equipment and pipeline manufacturing. This is achieved by applying the CO_2_ emission intensity derived from unit equipment, as delineated in Eqs. (2)–(4).

2 $$ C_{production} = C_{equipment} + C_{pipeline} $$

3 $$ C_{equipment} = \sum\limits_{j} {\sum\limits_{i} {\left( {1 + \varepsilon_{i} } \right)\left[ {M_{i} \omega_{ci} \left( {1 - \delta_{i} } \right) + M_{i} \omega_{ci}^{r} \delta_{i} } \right]} } + \sum\limits_{j} {M_{j} E_{j} ^{\prime}\omega_{e} } $$

4 $$ C_{pipeline} = \sum\limits_{j} {\sum\limits_{i = 1}^{3} {k_{j} \left( {1 + \varepsilon_{i} } \right)\left[ {M_{i} \omega_{ci} \left( {1 - \delta_{i} } \right) + M_{i} \omega_{ci}^{r} \delta_{i} } \right]} } + \sum\limits_{j} {k_{j} M_{j} E_{j} ^{\prime}\omega_{e} } $$

_Gt_.

$C_{equipment}$ and $C_{pipeline}$ refer to the total amount of CO_2_ emissions in the comprehensive production stage of system equipment and pipelines, respectively, in the units of kg. $\omega_{ci}$ and $\omega_{ci}^{r}$ refer to the CO_2_ emissions from the original production and recycling process of the *i*-type component material of the *j*-type equipment or the *j*-type pipeline per unit mass or volume, respectively. Within the context of material CO_2_ emission intensity, the unit is expressed in kilograms per unit mass (kg/kg) or kilograms per unit volume (kg/m^3^). $E_{j}{\prime}$ refers to the electric energy consumed by the second processing of the equipment of type *j* or the pipeline of type *i* per unit mass, the unit is *k* Wh/kg. $\omega_{e}$ refers to the CO_2_ emission factor of electricity, the unit is kg/kWh. $k_{j}$ refers to the number of pipes of type j. $\varepsilon_{i}$ refers to the abandonment coefficient of the *i-*th component material of the pipeline. $M_{i}$ refers to the mass of the constituent material of the first kind of pipe of the first kind of pipe of the *j-*th kind, the unit is kg. $\delta_{i}$ refers to the recovery coefficient of the i-component material of the *j*-type pipeline.

During the installation and construction phase, the primary source of CO_2_ emissions stems from the combustion of power energy during the operation of mechanical equipment and the release of materials essential for the construction of directly buried pipelines^30–32^. The calculation equations for this stage are presented in Eqs. (5–9):

5 $$ C_{construction} = C_{ct} + C_{ce} + C_{cp} + C_{cpm} $$

6 $$ C_{ct} = \sum\limits_{j} {\sum\limits_{i} {M_{i} L_{ij} \omega_{tj} \mu_{1} \lambda_{n} \left( {1 + \beta_{n} } \right)} } $$

7 $$ C_{ce} = \sum\limits_{k} {Q_{ck} \omega_{cek} \lambda_{n} \left( {1 + \beta_{n} } \right)} $$

8 $$ C_{cp} = \sum\limits_{m} {\sum\limits_{n} {k_{j} E_{mn} N_{mn} \omega_{en} \lambda_{n} \left( {1 + \beta_{n} } \right)} } $$

9 $$ C_{cpm} = \sum\limits_{j} {\sum\limits_{i} {k_{j} \left( {1 + \varepsilon_{i} } \right)\left[ {M_{pi} \omega_{ci} \left( {1 - \delta_{i} } \right) + M_{i} \omega_{ci}^{r} \delta_{i} } \right]} } $$

$\lambda_{n}$ refers to CO_2_ emission per unit fuel *n* consumed by mechanical operation, and the unit is kg/MJ. $\beta_{n}$ refers to the fuel consumed by mechanical operation, and CO_2_ generated by unit fuel in the upstream mining production stage. $\omega_{tj}$ refers to the energy consumption per unit turnover of the *j* mode of transportation of equipment *i*, MJ/t km. $L_{ij}$ refers to the transportation distance of the *i*-type equipment under the *j*-type transportation mode, km. Highway transportation is considered to be multiplied by the no-load factor of $\mu_{1}$ = 1.5. Other transportation modes are $\mu_{1}$ = 1, and $Q_{ck}$ refers to the engineering quantity of the* k*-type installation process of the machine room or heat exchange station. $\omega_{cek}$ refers to the energy consumption per unit quantity of the *k* installation process, MJ. $E_{mn}$ refers to the machine-team consumption of the nth kind of energy consumed by the *m* kind of construction machinery in the construction process of installing a first kind of pipeline, kg/ machine-team. $N_{mn}$ refers to the usage of *m*-type construction machinery in the process of installing a *j-*type pipeline. $\omega_{en}$ refers to the conversion coefficient of the *n-*th energy source, MJ/kg. $M_{pi}$ refers to the consumption of material *i* in the construction of a *j*-type pipeline, in kg or m^3^. In the operation and maintenance stage, CO_2_ emission is mainly generated by energy consumption, as shown in Eq. (10):

10 $$ C_{operation} = \sum\limits_{j = 1}^{{T_{l} }} {\sum\limits_{i} {Q_{ji} \omega_{oi} \left( {1 + \alpha_{i} } \right)AB} } $$

*A* refers to the carbon emission coefficient of energy, kgC/MJ. *B* refers to the carbon oxidation factor of energy combustion. $T_{l}$ refers to the system life. $Q_{ji}$ refers to the consumption of the *i*-type energy in the operation stage of the *j*-th year of the system life cycle, kWh. $\omega_{oi}$ refers to the average low calorific value of the *i-*th energy source, MJ/kg. $\alpha_{i}$ refers to the energy consumed by unit energy in the upstream mining, transportation, production and other processes of the *i-*type energy.

In the demolition treatment stage, CO_2_ emission is mainly shown in Eq. (11):

11 $$ C_{disposal} = \sum\limits_{i} {Q_{di} \omega_{di} \lambda_{n} \left( {1 + \beta_{n} } \right)} + \sum\limits_{j} {\sum\limits_{i} {M_{i} L_{ij} \mu_{1} \omega_{dj} \lambda_{n} \left( {1 + \beta_{n} } \right)} } $$

$Q_{di}$ refers to the engineering quantity when the *i-*type equipment in the machine room or heat exchange station is dismantled. $\omega_{di}$ refers to the energy consumption per unit quantity when the *i*-type equipment is dismantled, MJ.

Therefore, the total carbon emission $C_{all}$ in the life cycle of PTTP is as shown in Eq. (12):

12 $$ C_{all} = C_{production} + C_{construction} + C_{operation} + C_{disposal} $$

The various stages and steps in the process of carbon emissions are outlined as follows:

1. Power Pipeline Equipment Production Stage: This stage primarily encompasses the manufacturing and production of power pipeline equipment required for PTTP. It includes the production of key equipment such as transformers, cables, and switchgear. Carbon emissions during production primarily result from energy usage, raw material extraction, and processing, as well as emissions during industrial production.
2. Installation and Construction Stage: This phase involves the actual construction and installation processes of PTTP, including civil construction, equipment installation, and wiring. Sources of carbon emissions during construction include energy consumption, transportation, the use of mechanical equipment, and waste disposal.
3. Operation and Maintenance Stage: Once the PTTP is completed and operational, the long-term operation and maintenance become the core focus of this stage. Factors contributing to carbon emissions during this phase include energy consumption during equipment operation the use of mechanical equipment in maintenance processes, repairs, and replacements. The efficiency of equipment operation, regular maintenance, and updates are crucial for reducing carbon emissions during the operational period.
4. Dismantling and Disposal Stage: After the end of the lifespan of the PTTP, it enters the dismantling and disposal stage. This phase involves the dismantling of equipment and waste disposal, where the dismantling process and waste and residue disposal may lead to additional carbon emissions. The technologies and methods employed during the dismantling and disposal process directly impact the carbon emissions during this stage.

Understanding the carbon emission sources and key factors in each of these stages is crucial for comprehending the overall life cycle carbon emissions of PTTP. Considering the emissions sources and control factors at each stage is of significant importance in formulating strategies and measures to reduce carbon emissions.

### Experimental analysis

In this study, the PTTP within the new energy system of S city is employed as the primary data source. In order to assess the efficacy of the short-term user load forecasting scheme based on multi-task learning, the TensorFlow platform is utilized for simulation. Concurrently, various modules in Python are employed. The specific superparameter settings encompass a batch size of 100 and 100 iterations. The loss function is optimized using the random gradient descent algorithm, with the initial learning rate set at 0.001. The algorithm proposed in this study is benchmarked against alternative schemes based on MAE and prediction accuracy. These include:

1. Utilizing BiLSTM^33^, historical load, and future temperature data are input into forward and reverse networks separately.
2. Employing LSTM1^34^, historical load and future temperature data are input into the LSTM network simultaneously.
3. Using LSTM2^35^, only historical load data is input into the LSTM network.
4. Implementing the hybrid LSTM optimization algorithm proposed by Bacanin et al. (2023) for predicting the load of multi-energy consumption in power engineering. Furthermore, the carbon emissions of PTTP, based on LCA, are calculated in this study from three perspectives: total carbon emissions, annual carbon emissions per unit area, and the percentage of the total life cycle.

## Results and discussion

In this section, the performance of the constructed model and the experimental results are analyzed in depth and detail. This section will explain the effectiveness and applicability of the model, as well as the significance of the experimental results. The discussion of these results will help to better understand the advantages and potential improvement space of the model.

### Prediction performance analysis of different model algorithms

**Figure 4:**
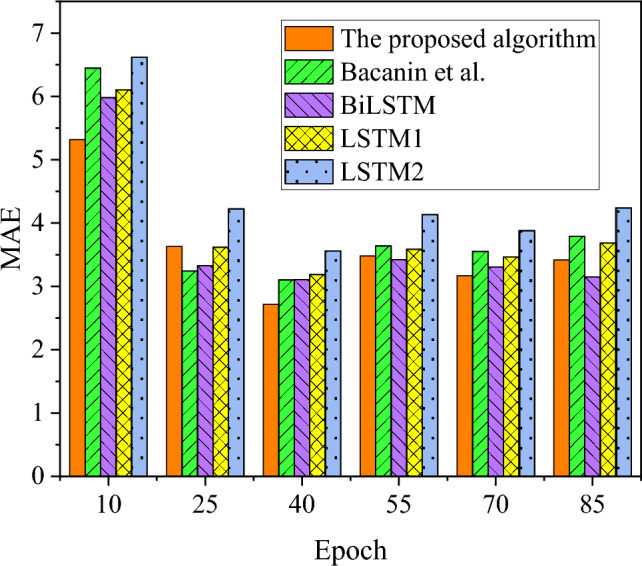
MAE results of different algorithms.

**Figure 5:**
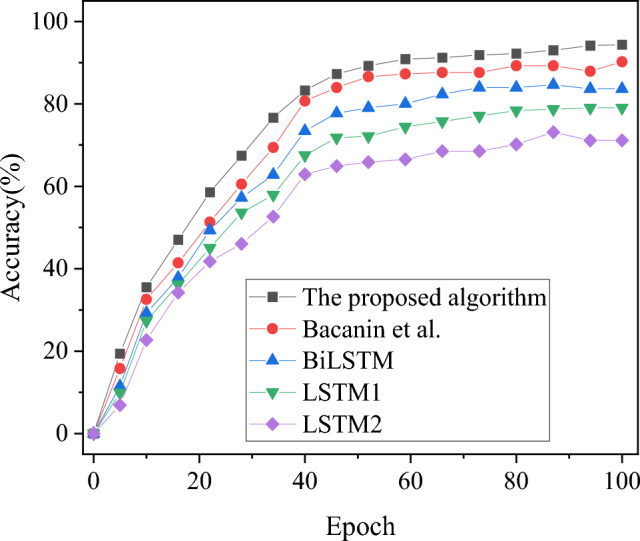
Power load forecasting accuracy results of different algorithms.

The algorithm presented in this study is juxtaposed with the algorithms proposed by BiLSTM, LSTM1, LSTM2, and Bacanin et al. (2023), evaluating their performance in terms of MAE and prediction accuracy. The comparative results are illustrated in Figs. 4, 5. The summary results of the prediction performance of each algorithm are shown in Table 4.

**Table 4:** Comparison of prediction performance of each algorithm.

Index	The proposed algorithm	Bacanin et al	BiLSTM	LSTM1	LSTM2
Accuracy (%)	94.34	90.22	83.64	79.04	71.14
MAE	3.62	3.96	3.71	3.94	4.44
F1-value (%)	90.71	86.93	82.32	76.40	70.81

In Figs. 4, 5, and Table 4, a comprehensive analysis of MAE and accuracy metrics is conducted to assess the performance of the algorithm model introduced in this study in comparison to models proposed by BiLSTM, LSTM1, LSTM2, and Bacanin et al. (2023). The results reveal a distinct advantage for the model algorithm presented herein, with a significantly lower average MAE of 3.62, as opposed to the higher average MAE exceeding 3.71 associated with algorithms devised by other researchers. The hierarchy of recognition errors, in terms of MAE, follows the order: model algorithm < Bacanin et al. (2023) < BiLSTM < LSTM1 < LSTM2. Furthermore, the power load forecasting accuracy of the model algorithm in this study notably outperforms other model algorithms, reaching 94.34%. Simultaneously, the comparative analysis of the F1-value reveals that the power load forecasting results achieved by the model algorithm presented in this study have attained 90.71%, surpassing the predictions of other model algorithms, all of which fall below 87%. Consequently, in comparison to algorithms proposed by other scholars, the short-term user load forecasting scheme based on multi-task learning outlined in this study exhibits heightened accuracy in power load forecasting. This enhancement contributes to the improved distribution of power load within the PTTP, providing more precise support for the low-carbon intelligent development of intelligent sustainable energy systems.

### Analysis of carbon emissions at different stages

**Figure 6:**
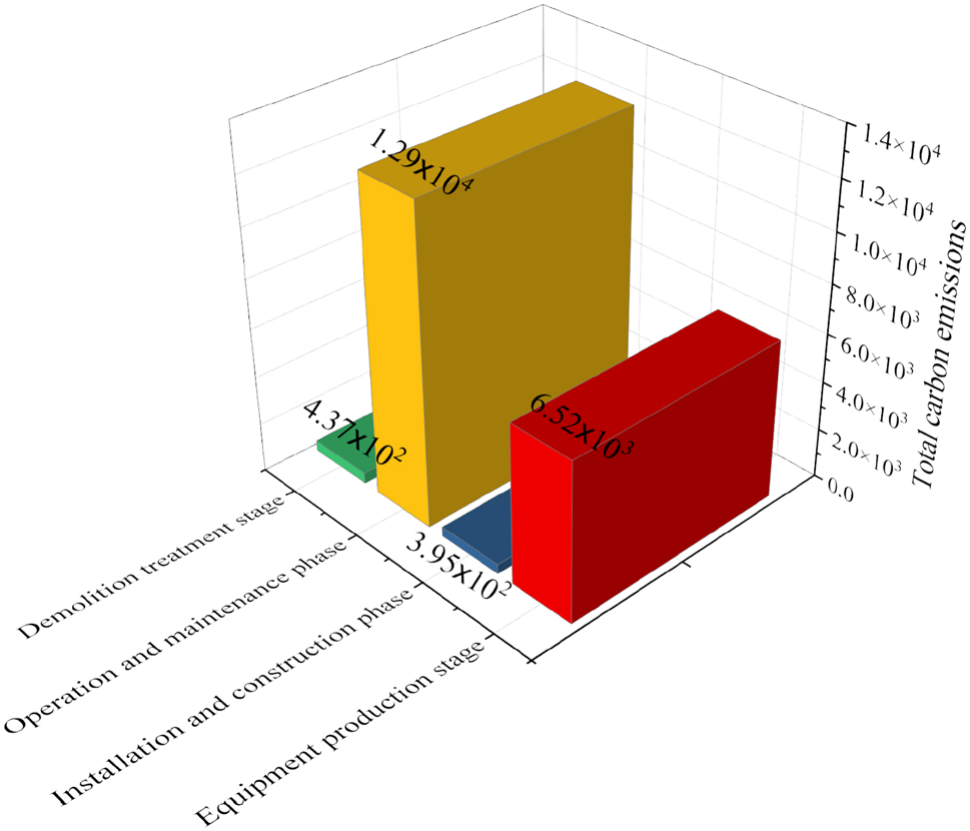
Results of total carbon emissions at each stage.

**Figure 7:**
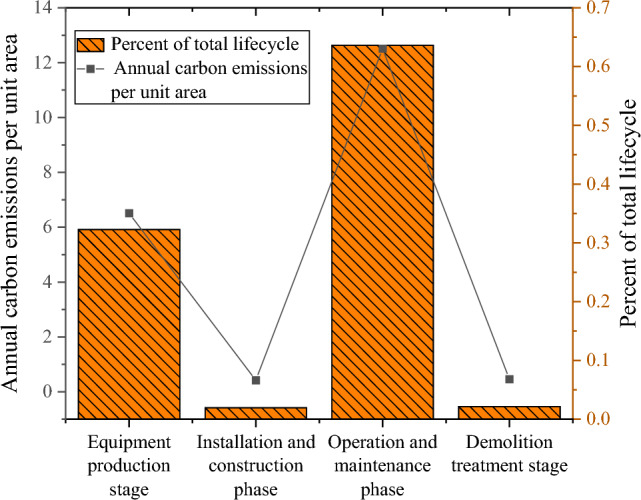
Results of total carbon emissions at each stage.

The carbon emissions of the four stages are analyzed, as shown in Figs. 6, 7.

In Fig. 6, the outcomes pertaining to total carbon emissions across the four stages of power pipeline equipment production, installation and construction, operation and maintenance, and demolition treatment indicate that the highest total carbon emissions occur during the operation and maintenance stage, amounting to 1.29 × 10^4^ t CO_2_ eq. Subsequently, the total carbon emissions during the equipment production stage are 6.52 × 10^3^ t CO_2_ eq. Notably, the installation and construction stage registers the lowest total carbon emissions, standing at 3.95 × 10^2^ t CO_2_ eq. Upon examining the results in Fig. 7, it becomes evident that the annual carbon emission per unit area is highest during the operation and maintenance stage, followed by the total carbon emissions during the equipment production stage. The installation and construction stage exhibits the lowest total carbon emissions, with a minimum value of 0.42 kg CO_2_ eq/ (a m^2^). Additionally, the summation of the percentages across the four stages in the total life cycle attains 100%. Consequently, with the adoption of a unified calculation method, an analysis of various influencing factors is undertaken, employing the concept of controlling variables in each stage. This analytical approach aims to identify the primary influencing factors of carbon emissions in power engineering, facilitating the control of these factors and the derivation of an optimized, energy-efficient intelligent power load scheme.

### Discussion

This study employs methodologies such as data collection, LCA, and model construction to validate the hypotheses presented. By collecting actual carbon emission data from different stages, conducting a LCA to analyze carbon emissions at each stage, and utilizing models to predict power loads, the proposed model algorithm in this study is compared with algorithms proposed by other scholars (BiLSTM, LSTM1, LSTM2, and Bacanin, among others) in terms of Mean Absolute Error (MAE) and prediction accuracy. The results indicate that the average MAE of the proposed algorithm in this study is significantly lower, at 3.62, compared to other algorithms with an average MAE higher than 3.71. Furthermore, the power load prediction accuracy of the model algorithm in this study is notably higher, reaching 94.34%. This suggests that the short-term user load forecasting scheme based on multi-task learning proposed in this study can provide more accurate support for the low-carbon intelligent development of intelligent sustainable energy systems. This aligns with the perspective of Morais et al.^36^, emphasizing the potential to enhance reliable energy distribution and achieve low-carbon intelligent development goals through the proposed forecasting scheme.

Additionally, an analysis of carbon emissions at different stages reveals that in the four stages of power pipeline equipment production, operation, and maintenance exhibit the highest total carbon emissions, followed by the equipment production stage, while the installation and construction stage shows the lowest total carbon emissions. This finding supports the hypothesis and identifies key factors in reducing carbon emissions in energy systems, aiding in the formulation of control strategies to minimize environmental impact and guiding the decarbonization of intelligent energy systems. Consistent with the views of Hamid et al.^37^, Fetisov et al.^38^ and Wang et al.^39^,this emphasizes the critical role of controlling and reducing carbon emissions in the operation and maintenance stage, necessitating the introduction of more energy-efficient and environmentally friendly methods and technologies during maintenance processes.

These findings can also serve as a reference for other fields, such as industrial production and the construction industry. The methodology presented is applicable to power load forecasting and can be extended to other domains requiring accurate prediction and control of energy consumption. By adopting similar models and algorithms, other industries can optimize energy utilization, reduce carbon emissions, and promote more sustainable development.

## Conclusions and prospects

This study commences by introducing the BiLSTM algorithm for intelligent power load prediction, establishing a short-term user load forecasting scheme grounded in multi-task learning. Employing the K-means clustering algorithm facilitates the classification of power load curves among a substantial number of residential users. Employing the LCA method, the life cycle of PTTP is meticulously delineated into four phases: equipment production, installation and construction, operation and maintenance, and demolition. Rigorous experimental evaluation reveals a significantly reduced average MAE of the model algorithm (3.62%) and markedly enhanced power load forecasting accuracy (94.34%). Additionally, the model demonstrates efficacy in quantifying carbon emissions across each life cycle stage, offering valuable insights for the prospective low-carbon intelligent development of sustainable energy systems.

However, this study still has some limitations. The acquisition of carbon emission data may be constrained by data availability or completeness, and certain assumptions within the model may not cover all scenarios, leading to a degree of limitation in the results. These constraints hinder a comprehensive understanding of the overall carbon emissions landscape in PTTP. Therefore, future studies could seek more comprehensive data sources, including additional on-site data and long-term monitoring. Moreover, refining the model and considering more factors may be focal points for future improvements. This could involve validating the model's accuracy and reliability by including more on-site case studies. Additionally, exploring low-carbon intelligent development requires considering more factors, such as the interactive influences of intelligent energy systems with social, economic, and policy aspects. Looking ahead, by comprehensively considering additional factors and data, the study can be further enhanced to provide more effective strategies and solutions for sustainable development.

## Data Availability

The data presented in this study are available on request from the corresponding author.
